# Diffusion Wave Spectroscopy Microrheological Characterization of Gelling Agarose Solutions

**DOI:** 10.3390/polym16182618

**Published:** 2024-09-16

**Authors:** Nuria Mancebo, Ramon G. Rubio, Francisco Ortega, Carlo Carbone, Eduardo Guzmán, Fernando Martínez-Pedrero, Miguel A. Rubio

**Affiliations:** 1 Departamento de Química Física, Facultad de Ciencias Químicas, Universidad Complutense de Madrid, Ciudad Universitaria s/n, 28040 Madrid, Spain; nmradio@ucm.es (N.M.); carlcarb@ucm.es (C.C.); eduardogs@quim.ucm.es (E.G.); fernandm@ucm.es (F.M.-P.); 2 Instituto Pluridisciplinar, Universidad Complutense de Madrid, Paseo Juan XXIII 1, 28040 Madrid, Spain; 3 Departamento de Física Fundamental, Facultad de Ciencias, Universidad Nacional de Educación a Distancia (UNED), Paso Senda del Rey 9, 28040 Madrid, Spain; mar@fisfun.uned.es

**Keywords:** agarose, diffuse wave spectroscopy, gelation, microrheology

## Abstract

This work investigated the gelation kinetics and mechanical properties of agarose hydrogels studied at different concentrations (in the range 1–5 g/L) and temperatures. Rheological measurements were performed by diffusing wave spectroscopy (DWS) using polystyrene and titanium dioxide particles as probes. The study emphasized the influence of gelation kinetics on the mechanical behavior of the hydrogels. The results showed that the gel properties were closely related to the thermal history and aging time of the samples. The insights gained from this study are critical for optimizing the performance of agarose hydrogels in specific applications and highlight the importance of controlling the concentration and thermal conditions during hydrogel preparation.

## 1. Introduction

Gels are ubiquitous in biological systems as well as in numerous technological fields including cosmetics, pharmaceuticals, food, smart materials, etc. [1,2,3,4,5,6]. For instance, many polymer solutions exhibit a sol–gel transition at high enough concentration and high or low enough temperatures [7,8,9,10]. In a very diluted sol state, these solutions behave as Newtonian fluids, while in the gel state, they exhibit a viscoelastic behavior characterized by a complex shear modulus, G*, which consists of two components: the real one, the elastic modulus G′, and the imaginary one, the loss modulus *G*″ such as G*(*T*,*ω*) = *G*′(*T*, *ω*)+*iG*″(*T*, *ω*). The shear modulus can be expressed as a function of the temperature, *T*, and the angular frequency, *ω*, and $i=\sqrt{-1}$. Although it is also pressure dependent, this dependence was not considered here because all of the experiments performed in this work were conducted at atmospheric pressure. As the system crosses from the sol to the gel states, *G*′ changes from being lower than *G*″ to exceeding it [11]. In contrast to the sol state, in the gel state *G*′(*ω* = 0) > 0.

Gelling systems are often subjected to variations in temperature and external mechanical perturbations of different frequencies. This requires knowing *G*′ and *G*″ at each *T* and *ω*. However, determining these values is challenging because the kinetics of the sol–gel transition are highly sensitive to *T* and the polymer concentration, *C*. In general, the transition occurs more rapidly at high *C* and at temperatures close to the transition point for a given concentration [12]. There are cases where the kinetics are relatively slow in comparison to the changes in *T* or the characteristic timescale of the mechanical perturbations, thereby allowing the state of the system to be little perturbed. Conversely, in other cases, the state of the system may undergo a significant alteration as a result of these external perturbations, leading to energy dissipation and subsequent effects on the temperature and mechanical properties of the gel [11]. All of the above makes it important to characterize G*(*C*,*T*, *ω*,*t*), *t* being the aging time (i.e., the time interval since the sample was prepared until G* is measured). Hereinafter, *t*_eq_ will be referred to as the equilibrium time, when *G*′(*C*,*T*,*ω*) and *G*″(*C*,*T*,*ω*) become time-independent.

**Scheme 1:**
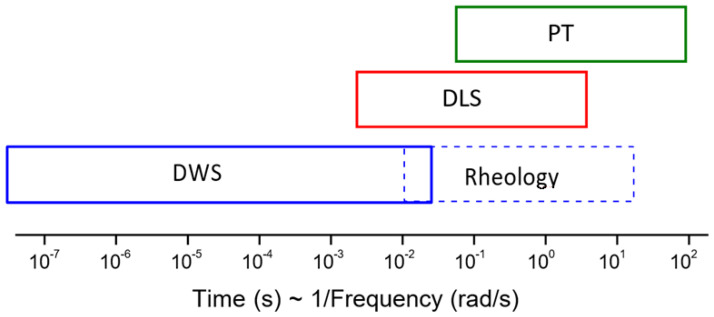
Summary of the frequency ranges that can be covered by rheological techniques: particle tracking by videomicroscopy (PT), standard dynamic light scattering (DLS), rheology using standard rotational rheometers, and diffusion wave spectroscopy (DWS).

A common experimental challenge is that the frequency *ω* range accessible to standard commercial oscillatory rheometers is typically narrow, usually between 0.001 and 30 Hz. This limitation frequently makes it difficult for a comprehensive study of *G*′ and *G*″ over the frequency at which the *T*_gel_ is placed, especially for low concentrations or temperatures far from *T*_gel_. In many cases, once *t*_eq_ is reached, the *G*′(*ω*) and *G*″(*ω*) curves at a specific *T* are used to construct a master curve using the *T*-*t* superposition method. However, this approach is only applicable to hydrodynamically simple systems, which are relatively uncommon [11]. Furthermore, this approach does not permit the examination of the temperature dependence of *t*_gel_ at a given concentration in gelling systems, making it necessary to use alternative techniques that can extend the *ω*-range. Microrheology is particularly well-suited for this purpose [13,14,15,16,17,18,19,20,21,22], as it has been demonstrated to be in agreement with rotational rheometer results in the overlapping frequency range, particularly in the case of highly homogeneous media [23,24]. A fundamental distinction between these two types of techniques is that rotational rheometers apply external strain to the sample, whereas passive microrheology relies on the dynamics of micro- or nanoparticles introduced into the system as tracers. In this work, the multiple scattered light from the sample was analyzed as a function of *C*, *T*, and *t*. Scheme 1 summarizes the characteristic times available for the different microrheological techniques, demonstrating that a combination of diffusion wave spectroscopy (DWS) and rotational rheology can cover a broad frequency range. However, it is important to note that the addition of external particles to the gelling system might alter the gel formation process if specific interactions occur between the surface of the particles and the polymer chains. This should be carefully avoided.

**Scheme 2:**
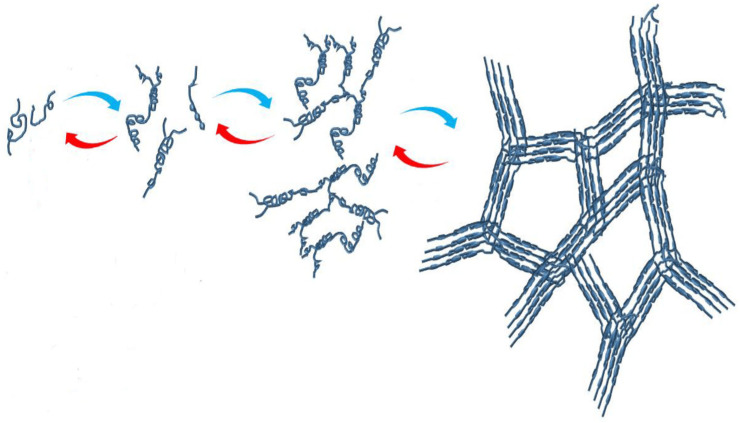
Qualitative steps during the gelling process (blue and red arrows indicate the cooling and heating steps, respectively). Adapted from reference [6], with permission under license Creative Common 4.0.

It should be noted that there are other microrheological techniques, the so-called active methods, that allow one to investigate the nonlinear viscoelastic regime: optical or magnetic tweezers [25]. These methods were not used in the present work. The aim of this study was to investigate the mechanical properties of physical gels, which are defined as gels in which polymer chains form nodes through physical interactions instead of through covalent bonds. An important feature of physical gels is that modifying *T* enables the reversible transition from the gel to the sol state by breaking the physical bonds. In this work, agarose solutions were selected for investigation due to their extensive use in food science and the absence of the strong ionic osmotic effects observed in polyelectrolyte gels, which simplifies the physics of the system. Furthermore, the physical process by which the gel is formed has been qualitatively understood and is illustrated in Scheme 2 [6]. On the other hand, the kinetics of gel formation has been found to be sufficiently slow to allow for the measurement of the mechanical properties at different aging times. The gelling process was studied over a wide range of frequency, temperature, and concentration ranges. Two chemically different microparticles and two particle sizes were used to assess the particle independence of the shear modulus of the system and to demonstrate the impact of specific chemical particle–polymer interactions.

### Theoretical Background

**Scheme 3:**
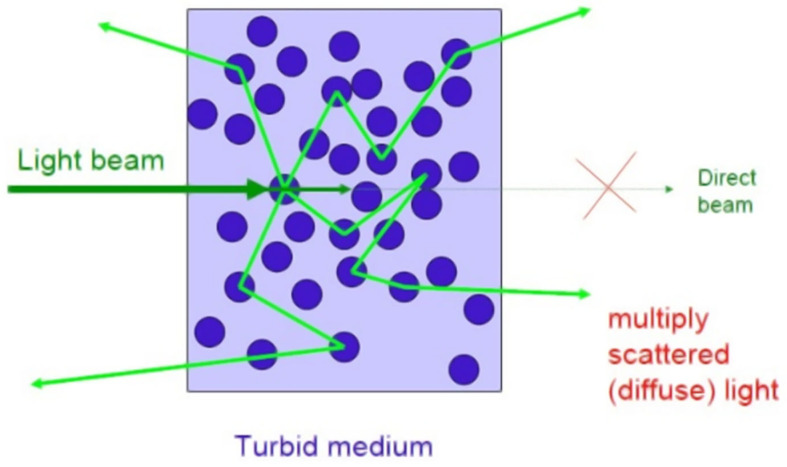
Mechanism of multiply scattering in a turbid sample. Photons must undergo a number of scatter events high enough by the particles to become diffusive.

The motion of particles in turbid, dense media gives rises to temporal fluctuations in the intensity of multiply scattered light. The dynamics of the scatters can be obtained from measurements of the temporal autocorrelation functions. In contrast to standard dynamic light scattering experiments, which require the absence of multiple scattering existing, the DWS technique is based on the opposite principle. Each photon must have undergone a sufficient number of scattering events to produce a diffuse light output, with scattering directions distributed randomly. This is illustrated in Scheme 3 [26]. For a DWS experiment, the intensity autocorrelation function in terms of the mean square displacement is given by [27]

(1) $$g^{\left(2\right)}\left(t\right)-1={\left(\int_{0}^{\infty }P\left(s\right)e^{-\frac{s}{l^{*}}k^{2}MSD}ds\right)}^{2},$$

where *k* represents the wave vector of the light in the medium. *P*(*s*) is the distribution of the path lengths of the photons in the sample, which is calculated with the diffusion model taking into account the experimental geometry. l* is the transport mean free path, that is, the length of the light trajectory before its direction is randomized. l* is given by

(2) $$l*=\frac{l}{\langle 1-\cos\theta \rangle },\;l=\frac{1}{\rho \sigma },$$

with *l* being the mean free path between scattering events, where *ρ* is the density of the sample and *σ* is the total scattering cross-section for a free particle in suspension. *MSD* accounts for the mean square displacement of the particles suspended in the system. It is important to note that the path length distribution is dependent on the size, shape, and interactions of the particles with the medium.

The necessity for a specific sample thickness in diffusing wave spectroscopy (DWS) ensures that virtually no unscattered light is transmitted through the sample. For the diffusion approximation to be valid, the sample thickness must be several times larger than l*. In practice, the correlation functions employed in DWS offer good approximations when the ratio of *L*/l* is at least higher than five.

The diffusion approximation is employed to describe the random walk of the light within the medium, and *P*(*s*) can be obtained from the solution of the diffusion equation for the appropriate experimental geometry. In the case of an infinitely large scattering medium, where light is introduced at a specific point and detected at a distance *r* (much larger than l*), the *P*(*s*) can be defined in terms of a Gaussian distribution that results from the central limit theorem, and describes the cumulative effect of many independent scattering events,

(3) $$P\left(S\right)={\left(\frac{3}{4\pi nl{*}^{2}}\right)}^{\frac{3}{2}}\exp\left(-\frac{3r^{2}}{4nl{*}^{2}}\right)={\left(\frac{3}{4\pi nl{*}^{2}}\right)}^{\frac{3}{2}}\exp\left(-\frac{3r^{2}}{4l{*}^{2}}\right)\;;\;n=\frac{s}{l*},$$

with *n* representing the number of steps, and *s* the path length. This provides an equation that allows for the calculation of l* from the autocorrelation functions for suspensions of particles of known size,

(4) $$g^{\left(2\right)}-1=\beta \left[\frac{\left(\frac{L}{l*}+\frac{4}{3}\right){\left(k_{0}^{2}MSD\right)}^{\frac{1}{2}}}{\sin h\left\{\frac{L}{l*}+\frac{4}{3}\right\}{\left(k_{0}^{2}MSD\right)}^{\frac{1}{2}}}\right].$$

Using Equation (4), the mean square displacement (*MSD*) of particles can be numerically calculated from the measured autocorrelation function, *g^(2)^*(*t*). This provides valuable information into particle motion at scales much smaller than the particle diameter. Since the motion of the particles is driven by the thermal energy *k_B_T*, it follows a purely Brownian pattern, where *MSD* = *6Dt*, with *D* representing the diffusion coefficient. This is the simplest dynamic behavior that is typically observed in Newtonian fluids, where the Stokes–Einstein relation applies. In more complex viscoelastic fluids, such as gels, the particle dynamics become more complicated, often involving more than one diffusion coefficient. This type of behavior has also been observed in non-gelling systems through standard dynamic light scattering (DLS) [28]. In order to determine the mechanical properties of the systems from the MSD results, one can calculate *G*′(*ω*) and *G*″(*ω*) using the method proposed by Mason [29]. Mason introduced a generalized Stokes–Einstein equation that describes the viscoelastic spectrum in Laplace space, providing a way to link MSD results with the mechanical characteristics of the material according to,

(5) $$\tilde{G}\left(s\right)=\frac{k_{B}T}{\pi RsMSD^{2}\left(s\right)},$$

where *MSD*(*s*) represents the Laplace transform of *MSD*(*r*). This equation has been derived by calculating the ensemble-averaged velocity autocorrelation functions resulting from the generalized Langevin equation, Equation (6), in a viscoelastic medium using a local memory function that is consistent with energy equipartition and the fluctuation-dissipation theorem

(6) $$m\frac{dv\left(t\right)}{dt}=f_{R}\left(t\right)-\int_{0}^{t}\gamma \left(t-t^{\prime }\right)v\left(t^{\prime }\right)dt^{\prime },$$

where $f_{R}\left(t\right)$ defines the random forces acting on the particle, which comprise the contribution from both the direct forces between the particles and the stochastic Brownian motion. These forces result in acceleration or deceleration of the colloidal particles in random directions. Therefore, the ensemble average of the fluctuating force is null. The integral term represents the viscous damping of the fluid and incorporates a generalized time-dependent memory function *γ*(*t*). Energy stored in the medium gives rise to significant alterations in the temporal correlations of the stochastic forces acting on the particle at thermal equilibrium. In light of the fluctuation-dissipation theorem and the delta-function correlation of a purely viscous fluid, one can write

(7) $$f_{R}\left(t\right)f_{R}\left(t^{\prime }\right)=2I\gamma k_{B}T\delta \left(t-t^{\prime }\right),$$

where *I* is the unit matrix, and *γ* is the drag coefficient. Although $\tilde{G}\left(s\right)$ is a concise representation, the standard one is the shear complex modulus G*(*ω*), which can be obtained by substituting *s* = i*ω* and identifying G*(*ω*) =$\tilde{G}\left(s\right)$. Using Equation (8), the macroscopic viscoelasticity of the material G*(*ω*) can be obtained from the local response, assuming that the bulk stress relaxation has the same behavior than the local relaxations that affect the particle dynamics. The method consists in converting the MSD data using the following modified algebraic form of the generalized Stokes–Einstein relation (GSE) [29,30],

(8) $$G^{*}=\frac{k_{B}T}{\pi \cdot R\cdot MSD\cdot \Gamma \left[1+\alpha \left(\omega \right)\right]},$$

where the gamma function, Γ, is approached by Γ(1 + α) ≈ 0.457(1 + α)2 − 1.36(1 + α) + 1.90 [31], and α is the power law exponent describing the logarithmic slope of the MSD vs. t curve. Then, the moduli *G’* and *G”* are given by

(9) $$G’\left(\omega \right)=\left|G^{*}\left(\omega \right)\right|\cos\left(\pi \alpha \left(\omega \right)/2\right)\;G^{\prime \prime }\left(\omega \right)=\left|G^{*}\left(\omega \right)\right|\sin\left(\pi \alpha \left(\omega \right)/2\right).$$

Xu and Amin [24] have shown that the above method leads to a good agreement between the data obtained by DWS and standard rotational rheometers.

## 2. Materials and Methods

### 2.1. Sample Characteristics and Preparation

Low sulfate content agarose (<0.15%) was supplied by Sigma-Aldrich (Saint Louis, MO, USA) and was used as received without further purification. The solutions were prepared with filtered Milli-Q water obtained by an AquaMAX™-Ultra 370 Series multicartridge purification system (Young Lin Instrument Co. Ltd., Gyeonggi-do, Republic of Korea), presenting a resistivity higher than 18 MΩ∙cm and a total organic content lower than 6 ppm.

We studied agarose hydrogels with an agarose concentration in the range between 1 and 5 g/L (0.1–0.5% *w*/*v*). Gels were prepared following the boiling water bath method that consisted of three consecutive steps. First, the appropriate mass of agarose powder was dispersed in Milli-Q quality water at room temperature. The solution was placed in a magnetic stirrer for one hour in order for the agarose to become hydrated and reduce foaming. Second, the solution was heated to 100 °C, under continuous stirring, to achieve the complete dissolution of the agarose. Third, the solution was permanently kept at a temperature of, approximately, 15 °C above the gelation temperature to ensure that the experiments always started from the sol phase.

We used two different types of monodisperse spherical particles as probes for the DWS measurements: polystyrene particles (Invitrogen, Waltham, MA, USA) with diameters of 977 nm and 400 nm, respectively, and titanium dioxide particles (VP Disp. W 2730X from Degussa, Germany) of 127 nm. These were dispersed in Milli-Q water to obtain a suitable particle concentration (around 0.1% *w*/*w*) for the DWS measurements.

### 2.2. Microrheological Measurements

We performed the diffusing wave spectroscopy (DWS) experiments in the transmission geometry by means of an LS Instruments DWS system (Fribourg, Switzerland). The laser emission at 658 nm is focused by a system of mirrors and lens at the center of the sample cell. A polarization analyzer is placed before the detection system. Multiply scattered light is collected by an optical fiber, split by a fiber optic beam splitter, and directed to the high quantum efficiency avalanche photodiode (APD) modules operating in Geiger mode. These units provide digital pulses in accordance with the arrival time of individual photons to the detector. Finally, the photon counts are transferred to the correlator. The typical lag time range is 1 μs to 10 s.

### 2.3. Thermal History of the Samples

Gel properties of gelling systems at concentrations high enough are strongly dependent on the gelation kinetics, which, in turn, are strongly dependent on how close the sample is to the gelation point. In this work, particular care was taken to ensure that: (i) at each temperature, measurements were made until the time variation dependence became negligible, and (ii) the thermal history of the samples was as close as possible to each other. Each sample was prepared, maintained at 55 °C during 1 h, and then the measurements started.

## 3. Results and Discussion

### 3.1. Gelling Kinetics Effects on the Mechanical Behavior

**Figure 1:**
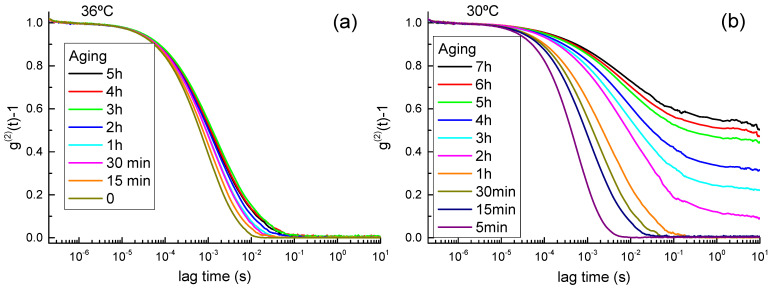
Time dependence of the ICF for an agarose concentration of 1 g/L and at 30 °C (**a**) and 36 °C (**b**) with PS particles 977 nm in diameter.

The kinetics of agarose gelation were studied for all samples. Figure 1 shows the evolution of the intensity autocorrelation function (ICF) at two different temperatures for a 1 g/L agarose concentration. At the highest temperature, the system’s IFC did not exhibit a significant evolution over the 7 h period, remaining in the liquid phase. However, at 30 °C, a significant evolution of the ICF was evident, demonstrating an increasing long-time correlation (*g*^(2)^(*t*) > 1) after a 2 h period.

It is worth noting that measurements using smaller colloidal beads as tracers—specifically, PS particles with a diameter of 400 nm instead of the 977 nm beads used in the experiment shown in Figure 1—yielded qualitatively similar results, indicating that the overall dynamics remained consistent despite changes in bead size. However, the reduction in bead diameter resulted in a systematic shift of the intensity correlation functions (ICFs) to shorter timescales across all tested conditions including concentration (*C*), temperature (*T*), and aging time. As shown below, the measurements obtained with both types of particles led to identical mechanical properties for the agarose solutions. Notice that the high values of *G*′ and *G*″, even at relatively low frequencies, indicate that the sample exhibited a viscoelastic behavior, which is consistent with the non-single exponential decay of the ICF curves shown in Figure 1b. This is reasonable given that, as will be demonstrated subsequently, the *T*_gel_ for this concentration was 30 °C.

### 3.2. Viscoelastic Behavior

**Figure 2:**
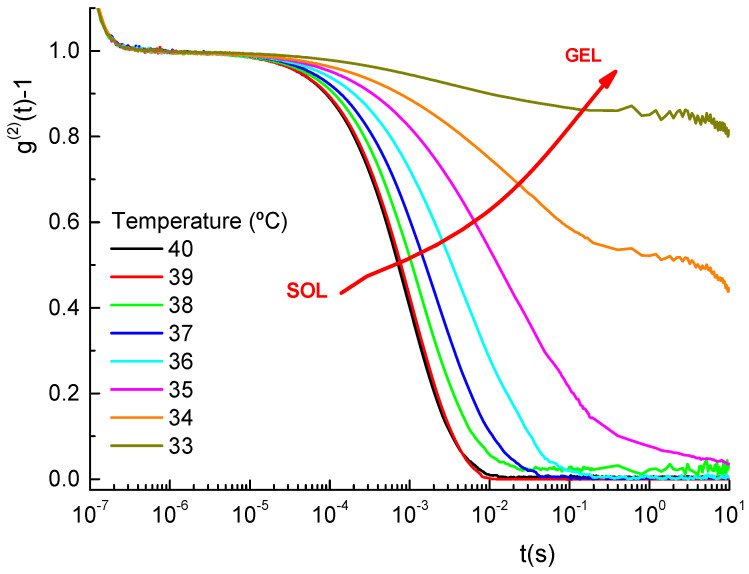
Temperature–decay time dependence of the ICF for agarose 3 g/L containing PS beads 977 nm in diameter as probes. The results for beads 400 nm in diameter were qualitatively similar.

Figure 2 shows the evolution of the DWS autocorrelation functions [*g^(2)^*(*t*) − 1], IFC, for the agarose system with a concentration of 3 g/L with polystyrene beads 977 nm in diameter (the results of the 400 nm diameter beads were qualitatively similar). At high temperatures, the ICF decayed exponentially, which is typical for a system that undergoes diffusion due to Brownian motion. As *T* decreased, the correlation functions no longer decayed to zero due to the slower motion of the clusters formed during the sol–gel transition [32].

**Figure 3:**
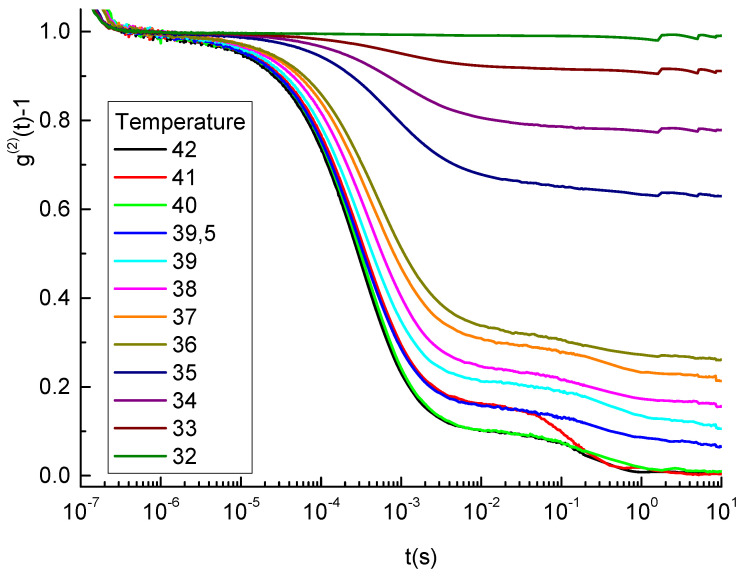
Temperature dependence of the ICF for agarose 3 g/L with TiO_2_ particles 127 nm in diameter.

Figure 3 shows the same conditions of the DWS technique and system but using titanium dioxide particles 127 nm in diameter as probes. In this case, a second decay was observed, even at high temperatures. This is typical in non-ergodic systems, and can be understood considering that the favorable interaction of the titania and the saccharide monomers make the particles stick to the gel nodes, where the density of saccharide groups is the highest [33].

In principle, one might expect that the differences observed are due to the lower size of the TiO_2_ particles that have a higher diffusion coefficient. In this way, long time features that cannot be observed with the PS particles would be observed with the TiO_2_ ones, however, we clearly show below that the situation is more complex.

**Figure 4:**
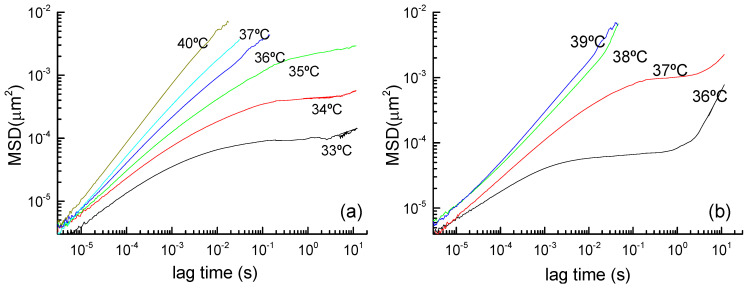
Temperature and lag-time dependences of the *MDS* of 977 nm PS latex probes in agarose solutions of 3 g/L (**a**) and 5 g/L (**b**) in the proximity of the gelling point.

For the sake of example, in what follows, the mean square displacement, *MSD*, curves will be shown only for the representative values of concentration and temperature. Figure 4 shows the mean square displacement curves for the 977 nm diameter PS beads dispersed into the agarose solutions of 3 and 5 g/L. At the highest temperatures, the *MSD* versus time had a linear dependence, as expected for single-exponential ICF. As temperature decreased, a subdifussive behavior was found at long times, followed by a plateau, and a further increase at higher times. Such behavior will be analyzed in detail below.

**Figure 5:**
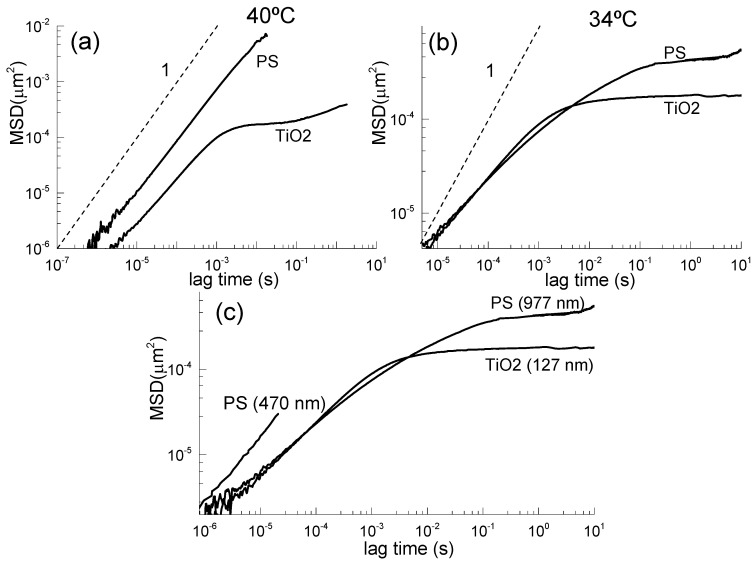
Lag-time dependence of the *MSD* for the 977 nm PS and the 127 nm TiO_2_ particles in agarose 3 g/L, at 40 °C (**a**) and 34 °C (**b**). In both panels, the dashed line represents the time dependence of the *MSD* corresponding to a purely Brownian process. (**c**) Comparative of the time dependence of the *MSD* for the 977 nm and 470 nm PS and the 127 nm TiO_2_ particles in agarose 3 g/L, at 34 °C.

It has been above-mentioned that the comparison of the *MSD* curves of the PS beads and the TiO_2_ ones is not compatible with the different sizes. Figure 5 shows the *MSD* curves using PS and TiO_2_ particles at 40 °C, corresponding to the liquid state of the agarose solution. The slope of the *MSD* curve had a value that was close to the one corresponding to a Newtonian fluid, whereas the results obtained when TiO_2_ particles were used showed the three regions found in viscoelastic systems, as found for both type of particles at 34 °C.

According to the Stokes–Einstein relationship [34], one should expect a higher diffusion coefficient for the smallest particles (i.e., the TiO_2_ ones), and therefore, the value of *MSD* for them should be higher than for the PS beads for a given lag time. This is the opposite to that shown in Figure 5, and the same was found for the other samples studied. Hurnaus and Plank [33] suggested that the Ti-OH groups of the TiO_2_ particles could strongly interact with the hydroxyl groups of the sugar units that form the backbone of the agarose chains through hydrogen bonds. This would decrease the energy of the gel when the particle maximizes the interactions with the sugar groups, which will happen when they are located close to the nodes of the gel (see Scheme 2), which in turn decreases their mobility. This result points to an obvious conclusion that deserves to be remarked: much care has to be taken when selecting the beads to perform microrheological experiments. Measurements using particles of different sizes and chemical nature are convenient for discarding the effect of strong specific interactions. A simple calculation to ensure that the motion of the particles of not highly affected by specific interactions with the polymer backbone is to test that the Stokes–Einstein relationship holds. In the present case, it was found that $\left(D_{977}/D_{470}\right)\approx \left(\frac{d_{470}}{d_{977}}\right)$ within the experimental uncertainties for conditions at which the mixtures behave as Newtonian fluids, hence indicating that the *MSD* curves reflect the shear properties of the systems.

### 3.3. Viscoelastic Models

To discuss the complex shear modulus G*(*ω*) of the gelling systems from the *MSD* curves, a rheological model has to be assumed. One model that predicts the behavior described above is Jeffrey´s, which corresponds to a Maxwell model in parallel with an elastic element.

Maxwell proposed a viscoelastic model from the non-Newtonian behavior of fluid, which consists of a viscous damper and an elastic spring connected in series [35]. For a Maxwell material, the modulus decomposition is:

(10) $$G*=G^{\prime }-iG”=G_{0}\frac{\omega^{2}\tau^{2}}{1+\omega^{2}\tau^{2}}-iG_{0}\frac{\omega \tau }{1+\omega^{2}\tau^{2}}.$$

From Equation (4), and using the Mason method, the *MSD* is given by Equation (5) [36],

(11) $$MSD=\frac{6K_{B}T}{\tau_{N}k\left(1+q\right)}\left(t+\frac{\tau_{M}q}{1+q}\left[1-e^{-\left(1+q\right)\frac{t}{\tau_{M}}}\right]\right).$$

The physical meaning in this Jeffrey scheme, the friction force is represented by two simultaneously operating mechanisms. One is responsible for viscous (Newtonian) friction with a zero-relaxation time, and the other is responsible for delayed (Maxwellian) friction with the characteristic time defined via a renormalized dynamic elasticity. Then, the Jeffrey rheological scheme is a phenomenological model of the complex medium that consists of two interacting continuum medium: simple viscous and viscoelastic fluids. This behavior may be expected for moderately concentrated polymer solutions in low molecular solvents and (with a correction for the enlarged scale of base units) from some micellar systems [37].

The Newtonian element has the response time *τ_N_*, while that of the Maxwellian element is *τ_M_*, with their ratio being *q* = *τ_M_*/*τ_N_*. Complete domination of one of the relaxation modes turns a Jeffrey into a Newtonian (*q* = 0) or a Maxwell (*q* = ∞) fluid. The elasticity and friction coefficients are introduced as *K* = 6π*GR*, where *G* is the elastic modulus and *R* is the radius of the particles.

**Figure 6:**
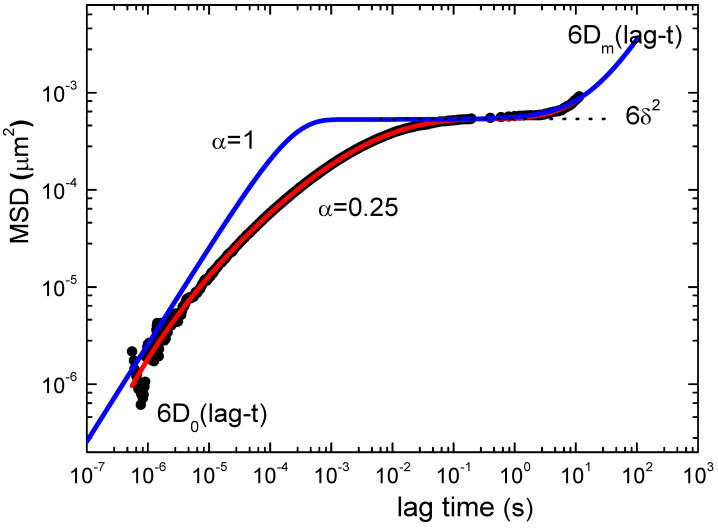
Curve of *MSD* as a function of lag time of PS latex in agarose 1 g/L. Blue line is a fit obtained with the Jeffrey Equation (11), so that α = 1. Red line corresponds to the best fit curve using the model Equation (12).

Figure 6 shows a set of experimental results together with the best fit to Jeffrey´s model (curve marked *α* = 1). Similar results were obtained for the rest of the samples. It is obvious that the extended Maxwell model was only able to fit the data at the lowest times (highest frequencies). This failure was found for all of the viscoelastic systems studied in this work. In order to overcome this problem, we introduced an ad hoc modification of the extended Maxwell model that led to [38]

(12) $$MSD=6\delta^{2}{\left(1-e^{-{\left(\frac{D_{0}}{\delta^{2}}t\right)}^{\alpha }}\right)}^{\frac{1}{\alpha }}\left(1+\frac{D_{m}}{\delta^{2}}t\right).$$

The exponent *α* is introduced following the Kohlrausch–Williams–Watts (KWW) idea of the stretched exponential, very frequently used to describe dynamic-mechanical and impedance spectroscopy data [5,39,40,41]. *D*_0_ and *D_m_* are the short time and long time diffusion coefficients, respectively, *δ* is the cage size, which is the amplitude of the motion, and is related to the plateau modulus *G*_0_ by [32]

(13) $$G_{0}=\frac{k_{B}T}{6\pi R\delta^{2}}.$$

It is possible to show that for *α* = 1, Equation (12) is equivalent to Jeffrey´s model. Figure 6 shows the best fit obtained with Equation (12). From the low- and high-time limits, *D*_0_ and *D_m_* can be obtained, respectively, and the plateau value corresponds to 6*δ*^2^. It must be remarked that a very good quality of fits was obtained using Equation (12) for all temperatures, concentrations, and for the three different particles. Of course, completely reliable values of *D_m_* and *δ* can only be obtained when both the plateau and the long diffusive branch are observed, whereas *D*_0_ requires a low time (high frequency data).

**Figure 7:**
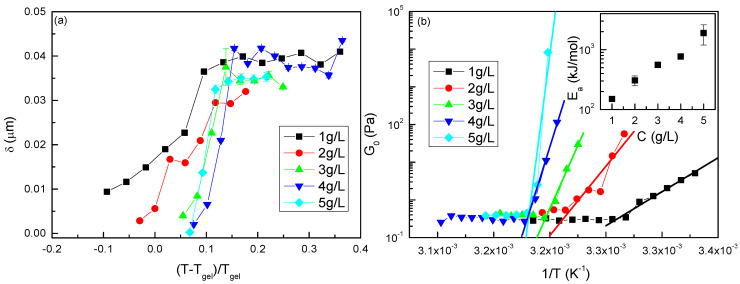
(**a**) Evolution of the cage size *δ* parameter involved in Equation (8) in the agarose solutions when the 977 nm PS particles were used. (**b**) Evolution of G_0_ with 1/T. The solid line represents the fit to the Arrhenius law. The inset displays the dependence of the apparent activation energy on the agarose concentration obtained from the fitting of the dependence of G_0_ on 1/T for concentrations below the *T*_gel_.

Figure 7 shows the evolution of the cage size *δ* that increases with *T* up to reaching a plateau that corresponds to the gel formation, which slows down the particle motion. It must be remarked that the values of *δ* were well below the size of the particles and were zero at temperatures close to the gel one. *δ* < *R* suggests that the motion of the particles does not map the detailed structure of the gel at such small scales but the macroscopic rheological properties. Even though the value of *δ* at the plateau was the same for all the concentrations studied, it was possible to build a single master curve using the temperature gel as the reducing parameter. Notice that the plateau modulus, G_0_ = *k*_B_*T*/(6π*Rδ*^2^), strongly increased as the temperature gel was approached from the sol state.

A quantification of the sol–gel transition can be conducted in terms of its temperature dependence using an empirical Arrhenius-like formalism [42,43]. Thus, assuming $G_{0}\propto A_{0}\exp(-E_{a}/RT)$, with $A_{0}$ being the pre-exponential factor and $E_{a}$ the activation energy, it is possible to fit the region corresponding to *T* < *T*_gel_, to obtain the activation energy (see inset in Figure 7b for the dependence of the $E_{a}$ on the agarose concentration). It should be noted that the activation energy obtained from the fitting presents an empirical character and cannot be considered equal to the activation energy of a Debye relaxation process [44]. The $E_{a}$ increases with the agarose concentration, as has been reported for agar-agar gels [42]. This allows ascribing the $E_{a}$ to the energy barrier associated with the formation of an agarose gel during the gelation process, and to the formation and fission of hydrogen bonds between agarose chains. Therefore, an increase in the agar concentration leads to the increase in the hydrogen bond interactions, and hence to the increase in $E_{a}$ [42].

**Figure 8:**
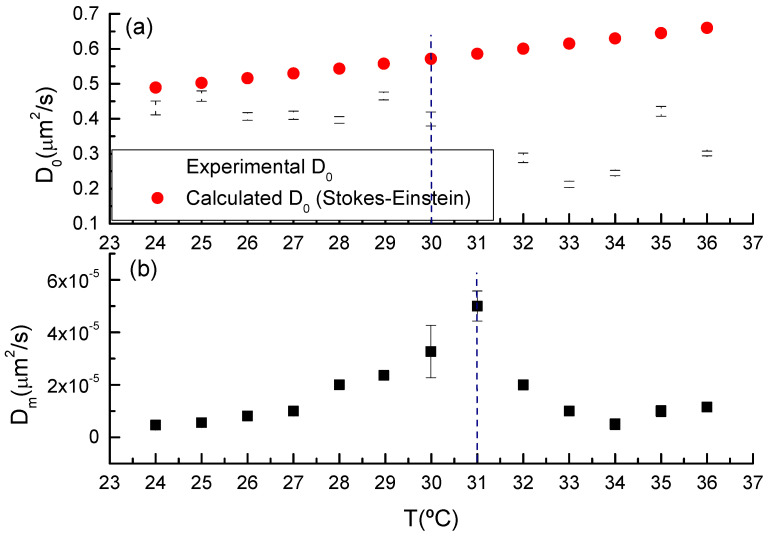
Evolution of the diffusion coefficients at short-time *D*_0_ (**a**) and long-time *D_m_* (**b**) involved in Equation (12), compared with the theoretical diffusion coefficient in water obtained by the Stokes–Einstein equation for *D*_0_, for an agarose concentration of 1 g/L.

Figure 8 displays the temperature dependence of the short- and long-time diffusion coefficients for PS beads in agarose solutions of 1 g/L. The results of *D*_0_ were quite close to those calculated from the Stokes–Einstein equation. This seems to mean that at the lowest temperatures, the short-time range of the *MSD* curve essentially corresponds to the Brownian motion of particles within the gel network cages. However, above the gel temperature, the particles move in a viscous polymer solution, and therefore *D*_0_ decreases. The behavior of *D_m_* presented a maximum at a temperature close to the *T*_gel_, and therefore, it is also expected that the long-time viscosity will. Such behavior looks like the one predicted by the mode-coupling theory used for describing the dynamics close to the glass transition [45], and has also been found in the dynamics of polymer quasi-2D monolayers [46]. As T increased from the gel state, the importance of the loss modulus increased and D_m_ increased. Above *T*_gel_, the sample became viscous, and D_m_ remained almost constant and close to zero until the sample approached the gel temperature and started to become viscoelastic. This agrees with the sharp increase in β and α that approaches unity right above *T*_gel_, which indicates that the rheological behavior approaches a Maxwell-type.

**Figure 9:**
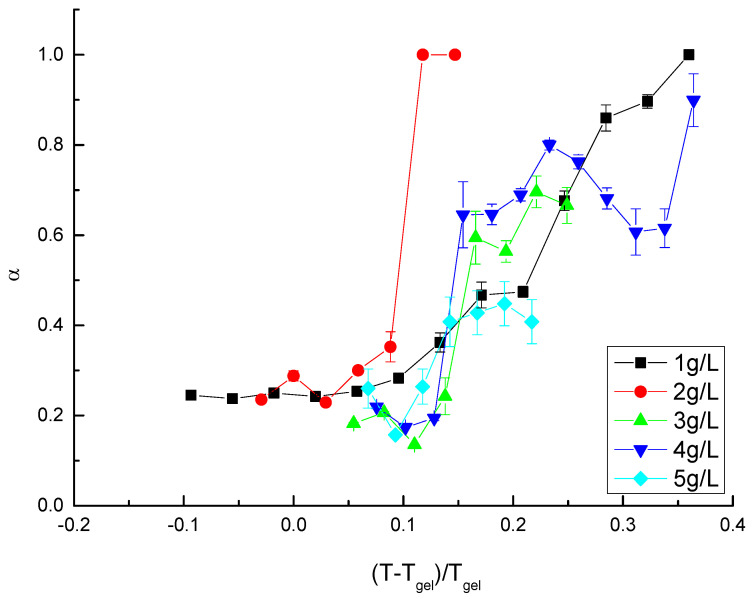
Temperature dependence of the exponent *α* parameter of Equation (5) in the agarose solutions using 977 nm PS particles.

The *α* parameter accounts for the width of the time relaxation distribution of the gel relaxation. To some extent, it is analogous to the beta parameter of the Kohlrausch–Williams–Watts (KWW) function frequently used to describe the glass transition [47]. Hence, one can expect values of alpha close to unity at high temperatures (near Maxwell-like relaxation) that decreases as the system approaches the gel temperature (Figure 9).

Values of *α* ≈ 0.25 corresponded to very broad time relaxation distributions. It is noteworthy that the high temperature values of *α* were below unity for agarose concentrations below 3 g/L, which is reasonable for viscoelastic polymer solutions.

### 3.4. Gel Temperature

**Figure 10:**
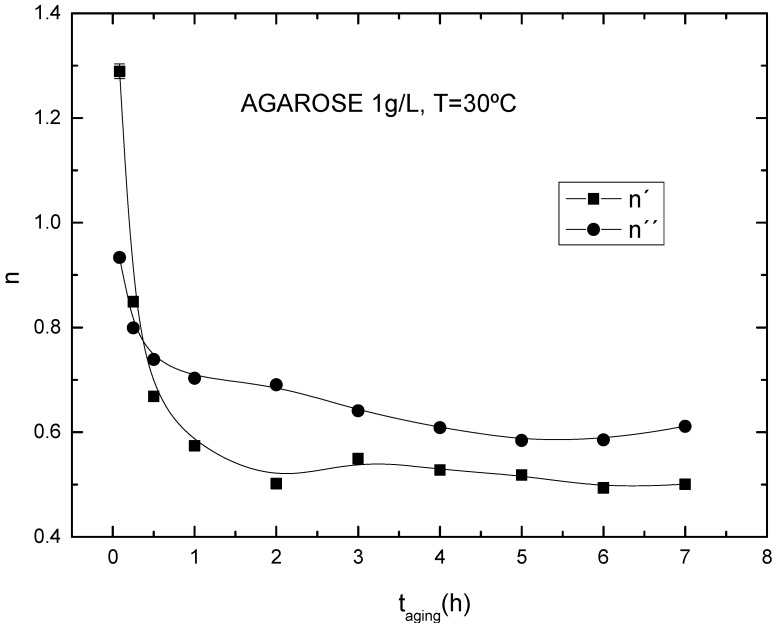
Power law exponents for agarose 1 g/L at 30 °C as a function of aging time.

According to the percolation theory of gelation [48], a power-law dependence of the elastic *G*′ and viscous *G*″ moduli on the frequency is expected from polymer systems near the point of the sol–gel transition (gelation point). As the system approaches the gelation point, the agreement with simple power law forms $G^{\prime }=G_{0}^{\prime }\omega^{n^{\prime }}$; $G^{\prime \prime }=G_{0}^{\prime \prime }\omega^{n^{\prime \prime }}$ are predicted, and the values of *n*′ and *n*″ approach each other as the gel point is reached. The percolation theory is applicable to aging time, and it predicts the equilibrium stage for the sample when both curves cross each other (Figure 10).

**Figure 11:**
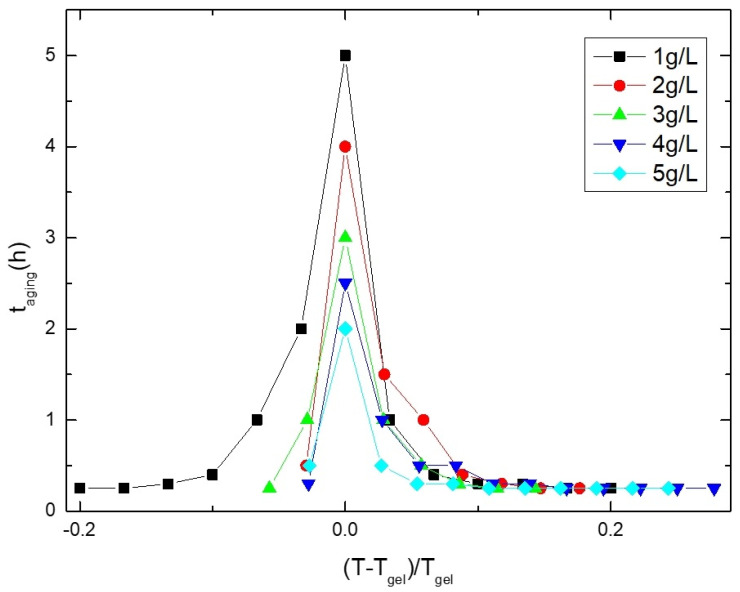
Effect of temperature on the agarose gelation kinetics.

Figure 11 shows the time needed for the equilibration as a function of *T* and *C*. As expected, that time increased as *C* decreased when approached from temperatures above *T_gel_*, and beyond that point, it decreased again.

**Figure 12:**
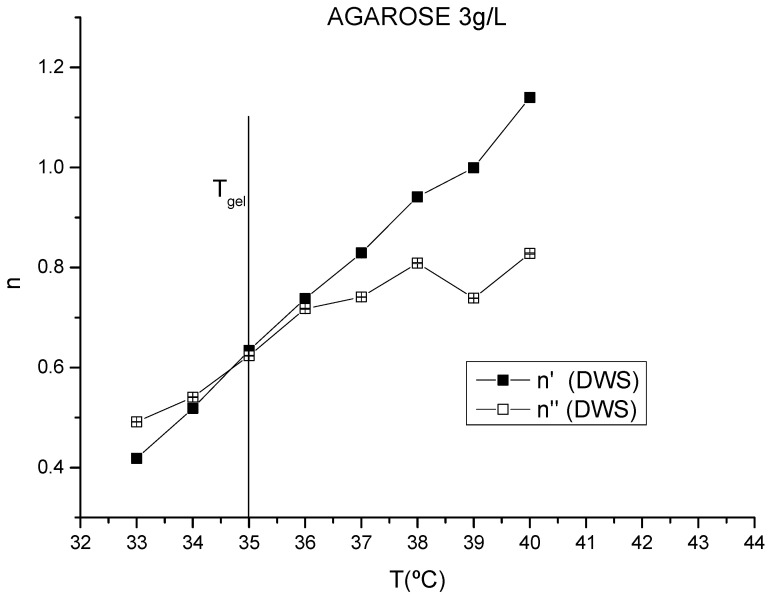
Power law exponents for 3 g/L agarose as a function of temperature. Note that the crossover of the values obtained by both techniques was the same.

As shown in Figure 12, agarose forms a gel when a homogeneous solution is cooled from a liquid to a temperature below the ordering temperature, which is the gel temperature, and depends on the polymer concentration. In the present case, it was observed that the gel temperature decreased gradually with the gel concentration. Most of the error bars were smaller than the symbols. Results of the power law exponents in the agarose concentration ranging from 1 to 5 g/L predict the gelation temperature from their crossover.

Winter and Chambon proposed a reliable criterion for the determination of the gel point temperature *T_gel_* based on the temperature dependence of the loss tangent, tanδ = *G*″/*G*′, at different frequencies [49]. According to the authors, at *T_gel_*, tanδ becomes frequency independent, and all the curves coincide at different oscillation frequencies. The values obtained were in agreement with the values reported in the literature [50]. For the 1 g/L sample, this temperature could not be determined without ambiguity due to the low concentration to form the gel.

**Figure 13:**
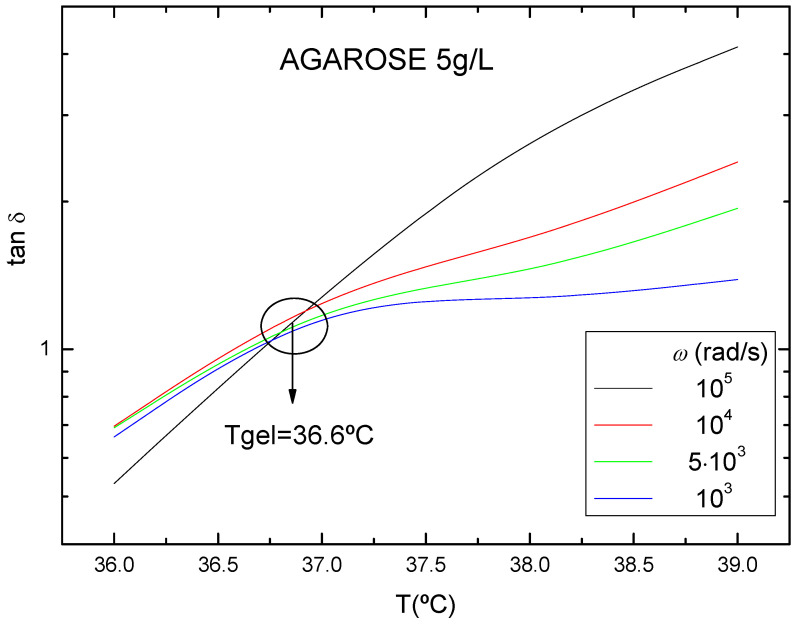
Temperature dependence of tanδ at different frequencies for C = 5g/L. The frequencies are given in the plot. Note that the *T* scale is quite expanded, thus the experimental uncertainty of *T*_gel_ can be considered within the circle.

**Figure 14:**
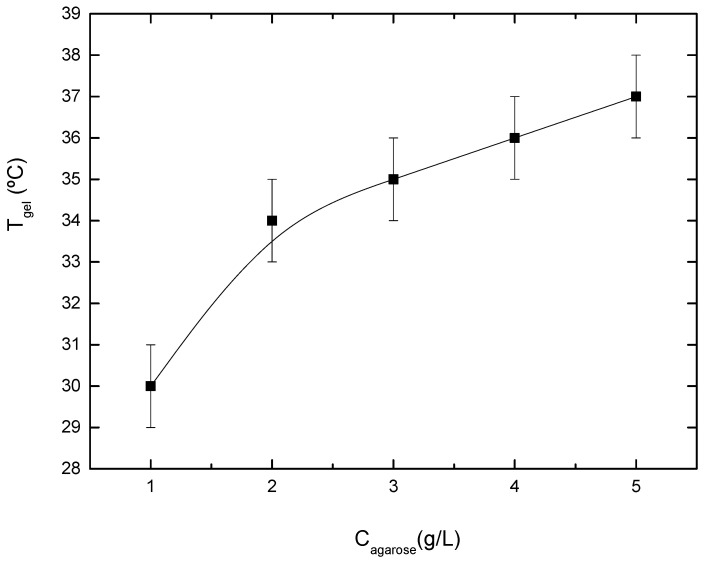
Concentration dependence of the agarose gelling temperature as obtained by microrheology (DWS) using percolation theory. The experimental error was of the order of ± 1 °C.

Figure 13 shows the results obtained for a concentration of 5 g/L agarose. The occurrence of a critical gelation temperature at which tan*δ* is *ω*-independent aligned remarkably with the value predicted by percolation theory. Similar results were found for the other concentrations, and the values obtained for *T_gel_* are shown in Figure 14.

## 4. Conclusions

This study has provided a comprehensive analysis of the gelling kinetics and mechanical properties of agarose hydrogels across a range of concentrations (1–5 g/L) and temperatures. By employing microrheological properties analysis through diffusing wave spectroscopy (DWS), it was possible to gain important insights into the behavior of these hydrogels under varying conditions.

The rheological measurements revealed that both the gelling kinetics and mechanical properties of agarose hydrogels are highly dependent on the concentration and temperature. Specifically, higher concentrations of agarose resulted in stronger gels, as evidenced by the higher dynamic moduli. This indicates that the polymer network becomes more robust with increased agarose content, enhancing the gel’s mechanical stability. Temperature, on the other hand, plays a crucial role in the gelation process. Lower temperatures facilitate the gelation process by promoting the formation of the agarose network, while higher temperatures tend to inhibit gel formation, delaying the gelling process and leading to weaker gels. Through DWS, we explored the microrheological properties of the hydrogels, providing a more detailed understanding of the internal dynamics of the gel network. The use of polystyrene and titanium dioxide particles as probes allowed us to monitor the motion within the hydrogel, further elucidating the impact of concentration and temperature on the gel’s microstructure. This technique demonstrates that gel properties are intricately linked to the internal structure and dynamics of the agarose network.

A key finding from this study was the significant influence of thermal history and aging time on the properties of agarose hydrogels. Samples that were subjected to different thermal histories exhibited notable variations in their mechanical properties, underscoring the importance of controlled thermal conditions during the preparation and storage of hydrogels. Similarly, the aging time of the samples was found to affect the gel properties, with longer aging times generally leading to more stable and robust gels. This aging effect is likely to be due to the continued restructuring and strengthening of the agarose network over time.

The transition from sol to gel state is a critical factor that influences the final properties of the hydrogel. By elucidating the factors that affect this transition, such as concentration, temperature, thermal history, and aging time, this research contributes to a deeper understanding of the fundamental processes governing the behavior of agarose hydrogels. These findings have important implications for the application of agarose hydrogels in various fields, particularly in biomedical and industrial contexts where the precise control over gel properties is crucial. The insights gained from this study provide a foundation for optimizing agarose hydrogels, ensuring that they meet the desired criteria for mechanical strength, stability, and performance

## Data Availability

Data are contained within the article.
